# Ocular Surface Infection Mediated Molecular Stress Responses: A Review

**DOI:** 10.3390/ijms23063111

**Published:** 2022-03-14

**Authors:** Samayitree Das, Sharon D’Souza, Bhavya Gorimanipalli, Rohit Shetty, Arkasubhra Ghosh, Vrushali Deshpande

**Affiliations:** 1 GROW Research Laboratory, Narayana Nethralaya Foundation, Bangalore 560 099, India; samayitreedas@narayananethralaya.com; 2 Cornea and Refractive Services, Narayana Nethralaya, Bangalore 560 010, India; drsharondsouza@gmail.com (S.D.); bhavya.g.2011@gmail.com (B.G.); drrohitshetty@yahoo.com (R.S.)

**Keywords:** host stress response, ocular surface infections, bacterial keratitis, fungal keratitis, viral keratitis, bacterial conjunctivitis, fungal conjunctivitis, viral conjunctivitis, proteins, gene expression

## Abstract

Infection mediated ocular surface stress responses are activated as early defense mechanisms in response to host cell damage. Integrated stress responses initiate the host response to different types of infections and modulate the transcription of key genes and translation of proteins. The crosstalk between host and pathogen results in profound alterations in cellular and molecular homeostasis triggering specific stress responses in the infected tissues. The amplitude and variations of such responses are partly responsible for the disease severity and clinical sequelae. Understanding the etiology and pathogenesis of ocular infections is important for early diagnosis and effective treatment. This review considers the molecular status of infection mediated ocular surface stress responses which may shed light on the importance of the host stress-signaling pathways. In this review, we collated literature on the molecular studies of all ocular surface infections and summarize the results from such studies systematically. Identification of important mediators involved in the crosstalk between the stress response and activation of diverse signaling molecules in host ocular surface infection may provide novel molecular targets for maintaining the cellular homeostasis during infection. These targets can be then explored and validated for diagnostic and therapeutic purposes.

## 1. Introduction

The ocular surface of the human eye comprises the cornea, conjunctiva, lacrimal glands, and eyelids [1] and is inhabited by several microorganisms due to constant exposure to the external environment. The cornea is a transparent, avascular tissue that acts as a structural barrier, protects the eye against infections and contributes two-thirds of the refractive power of the eye [2]. The corneal microstructure consists of epithelial cells, keratocytes, endothelial cells and an extracellular matrix consisting primarily of acellular collagen and glycosaminoglycans. Microbial pathogens can infect various layers of the cornea which leads to keratitis [3]. The conjunctiva is a thin, transparent tissue layering the outer surface of the eyeball (bulbar conjunctiva) and extending onto the inner surface of the eyelids (forniceal and palpebral conjunctiva). It is composed of a surface layer of non-keratinizing, stratified, squamous epithelium overlying a vascular stroma composed of loose connective tissue [4]. Infection of the conjunctiva by various pathogens can result in conjunctivitis [5].

The ocular surface has commensal organisms but the presence of certain opportunistic pathogens can result in florid infections with serious clinical consequences [6]. Depending on the structure involved in the infection, the patient can present with corneal ulcers (keratitis) or conjunctivitis when it involves the cornea or conjunctiva, respectively. Corneal ulcers of infectious etiology are a major cause of visual impairment globally and can be caused by bacteria, fungi, viruses, or protists [7]. The incidences of bacterial, fungal, or other microbial infections on the ocular surface vary greatly in numbers globally. The severity of infections can range from mild and non-visually significant to severe sight threatening corneal ulcers requiring intensive topical and systemic therapy and even surgical intervention [8]. Gram-positive infections (commonly caused by *Staphylococcus* or *Streptococcus* bacteria) are classically well-localized ulcers with comparatively less local surrounding inflammation, although in some cases, they can result in large corneal ulcers that require therapeutic corneal transplantation. Gram-negative (most commonly *Pseudomonas aeruginosa*) infections are more aggressive with dense corneal cellularity and suppuration with prominent keratolysis within the infiltrate [9]. They are also associated with more clinical inflammation than a Gram-positive infection. Fungal keratitis is usually a more indolent infection and can have a relatively longer clinical course. Classic features such as the feathery margins, satellite lesions, and dry appearance of the ulcer can guide clinical suspicion and diagnosis [9]. However, since this classic clinical appearance may vary based on other factors such as the patient’s systemic health and prior medications used, additional diagnostic tests are typically required for accurate diagnosis.

Conjunctivitis is a common condition affecting the ocular surface which can be infective or non-infective. The most common organisms causing infective conjunctivitis are viruses (e.g., *Adenovirus*) and bacteria (e.g., *Streptococcus pneumoniae*, *Haemophilus influenzae*, etc.) [10]. Depending on the organism involved, there can be various levels of inflammation. The microbial etiological profile depends on the geography, specific risk factors, occupational and economic background of the population [8]. Since the economic burden of corneal infections is high and the access to health care is relatively limited in developing countries, effective management of microbial keratitis and conjunctivitis is important to prevent sight threatening complications [11].

Understanding the etiopathogenesis and associated clinical features of the most common ocular surface infections has helped unravel the complexities in diagnosis and subsequent treatment of infectious eye disease [11]. An accurate microbiological diagnosis helps enable the clinician to formulate an effective treatment regime, taking into account the specific antimicrobial sensitivity patterns of the pathogens where possible. Microbiological techniques such as microscopic analysis of sample smears and cultures to isolate the infective organism remain the most commonly used methods of diagnosis. Additional techniques such as in vivo confocal microscopy show characteristic features in cases of *Acanthamoeba* and fungal ulcers and can aid diagnosis. Even though these techniques provide an accurate diagnosis in many cases, there are still certain cases where diagnosing the causative organism and poor host response to the treatment can be a challenge [12]. Particularly, in cases of complex co-infections with multiple organisms, which may happen particularly in tropical regions, it is challenging for standard techniques to identify causation objectively. In most cases of refractory infections, a better understanding of the host tissue response is a critical aspect that can help tune the treatment to optimize the outcomes. Thus, there remains a clinical need for alternative approaches to diagnosis and monitoring which can give us more accurate and reliable results to design effective treatment strategies.

Molecular advancements have paved a way to diagnose even those cases where organisms are difficult to culture or visualize by standard diagnostic techniques. High-throughput techniques have aided in the discovery of previously unknown networks based on protein interactions and cellular changes on a global scale. The study of gene regulation and expression facilitates the understanding of normal, abnormal, or pathological cellular processes in the host which are functionally associated with disease. Additionally, gene expression studies proved to be very important in interpreting the contribution of the transcriptome to immune dysregulation [13] and immune response [14].

In this review, we bring together all the molecular studies related to ocular surface infection (bacterial, viral, and fungal) mediated host cellular and stress responses to understand the current status of biological processes involved that may have potential relevance for diagnostic and therapeutic interventions. Our review also highlights the current diagnostic and treatment methods available and the challenges associated with them in different types of host ocular surface infections.

## 2. Clinical Presentation of Keratitis and Conjunctivitis and Treatment Modalities

**Figure 1:**
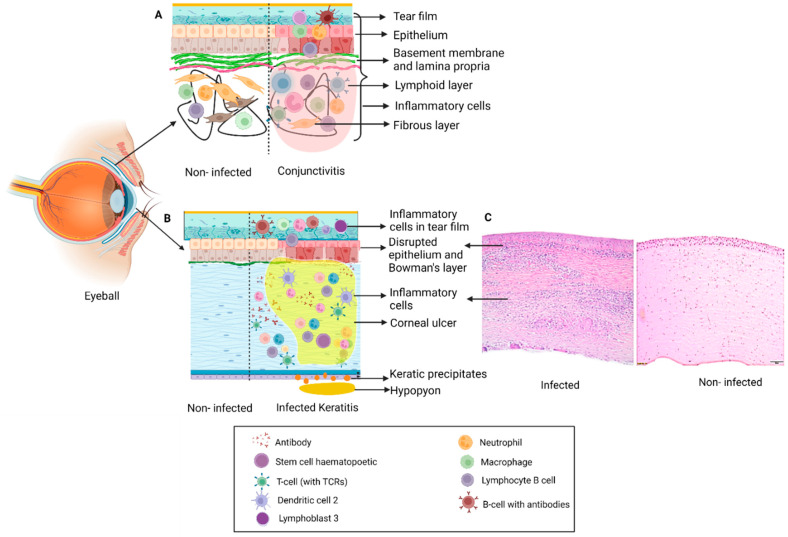
Schematic representation of changes in ocular tissue in: (**A**) Conjunctivitis: Inflammation of conjunctiva with infiltration of immune cells (neutrophils, lymphocytes, mast cells) which results in disruption in structure and inflammation leading to clinical symptoms and signs. (**B**) Keratitis: Infection of the cornea by different pathogens results in formation of a corneal ulcer or infiltrate with disruption in Bowman’s layer, epithelial and stromal edema with presence of inflammatory cells. Severe cases can also have inflammation in the anterior chamber in the form of hypopyon and keratic precipitates on the endothelium. (**C**) H&E staining of section on the right shows the histopathology of an infected cornea illustrating disruption of the Bowman’s layer and dense infiltration of the stroma by polymorphonuclear cells.

The ocular surface includes the cornea, limbus, and the entire conjunctival surface from superior lid margin to inferior lid margin [1]. Different types of keratitis and conjunctivitis can result in significant ocular discomfort and in severe cases can also impact vision (Figure 1, Table 1).

### 2.1. Keratitis

#### 2.1.1. Bacterial Keratitis

**Figure 2:**
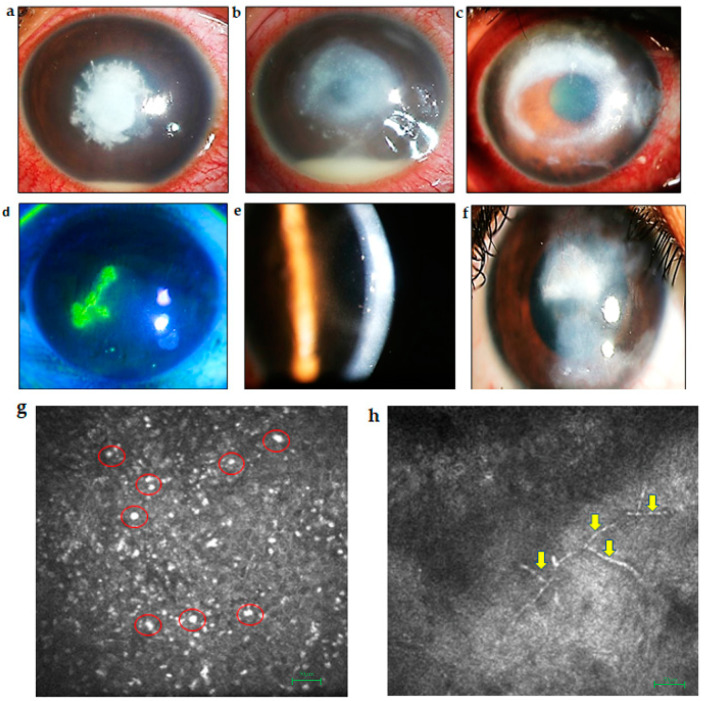
Slit-lamp photographs showing clinical features of different keratitis. (**a**) Fungal keratitis showing classic feathery margins and hypopyon. (**b**) Bacterial keratitis showing central dense infiltrate with hypopyon in the anterior chamber. (**c**) Acanthamoeba keratitis showing ring infiltrate in the cornea. (**d**) Herpes viral epithelial keratitis showing dendrite stained with fluorescein dye seen under cobalt blue filter. (**e**) Disciform keratitis shown with slit image of the cornea showing central stromal edema with keratic precipitates on the endothelium. (**f**) Recurrent herpes viral stromal keratitis with peripheral deep vascularization and central stromal edema and scarring. (**g**) In vivo confocal microscopy (IVCM) image of a cornea with Acanthamoeba keratitis showing hyperreflective cyst form of acanthamoeba and inflammatory cells (red circles). Panels shown are representative IVCM images with a depth of 31 microns. (**h**) IVCM image of a cornea with fungal keratitis showing fungal filaments (yellow arrows). Panels shown are representative IVCM images a depth of 320 microns. Confocal images were taken using Rostock Corneal Module/Heidelberg Retina Tomograph II (RCM/HRT2; Heidel Engineering GmBH, Dossenheim, Germany). Scale bar represents 50 µm.

Bacterial keratitis can have a varied clinical course which affects the central or peripheral cornea at different depths. Some of the common pathogens causing bacterial keratitis are *Staphylococcus aureus*, *Streptococcus pneumoniae* and *Pseudomonas aeruginosa* [15]. The disease process of bacterial keratitis initially goes through the stages of progressive infiltration and active ulceration from which it can progress and worsen in severity or start to regress and heal [16]. Corneal infections are rare in a healthy eye, however, altered defense mechanisms of the host cornea allow bacteria to invade the corneal epithelium. The severity of the disease is dependent on host immune response, the strain of the pathogen, the size of the inoculum, duration of the infection, and the antecedent therapy. Bacterial keratitis usually presents with symptoms of eye pain, blurred vision, photophobia, and eye discharge [17] (Figure 2b). The infiltration in the cornea is usually associated with an epithelial defect and stromal edema [17] which, if it worsens, can progress to corneal perforation, scleritis, and even endophthalmitis. Certain bacteria such as *Neisseria gonorrhea*, *Neisseria meningitidis* and *Corynebacterium diphtheriae* can also penetrate an intact epithelium [18]. Rapid diagnosis and initiation of the treatment are extremely important in preventing devastating vision loss. Slit-lamp examination shows the presence of corneal infiltrate which can be scraped and sent for microbiological analysis for the diagnosis [18]. Topical antibiotics are the mainstay of therapy especially in early cases but severe or recalcitrant infections may require systemic medications and in severe cases may also require surgical intervention with a therapeutic keratoplasty [19]. The choice of antibiotic depends on the suspected causative organisms and their sensitivity pattern. Fluoroquinolones, cephalosporins, and aminoglycosides are some of the commonly used medications [20].

#### 2.1.2. Fungal Keratitis

Fungal keratitis, also referred to as keratomycosis, is caused by the invasion of the cornea by pathologic fungi. Causative agents include septate filamentous fungi, i.e., *Fusarium* sp., *Aspergillus* sp., yeastlike organisms of *Candida* sp., and aseptate fungi such as *Rhizopus*. Fungal infections are classically indolent but can still result in severe sight threatening infections [17]. They are a major cause of vision loss in developing and tropical countries. Trauma with vegetative matter, chronic ocular surface diseases, diabetes, and contact lens usage can predispose to fungal keratitis [21]. Corneal ulcers caused by filamentous fungi typically present with dry looking infiltrates with feathery margins. They can also have satellite lesions, a hypopyon, and endoexudates. [21] (Figure 2a). Topical antifungals such as natamycin 5% and voriconazole 1% eye drops are commonly for the treatment of filamentous fungal keratitis. Topical amphotericin B 0.15% eye drop is an alternative, but needs to be reconstituted into a topical formulation from the injectable form and has more toxicity than the other medications [22]. Laboratory diagnostic methods include potassium hydroxide mounts to identify fungal filaments, growth in Sabouraud’s dextrose agar, and molecular analyses by PCR [17].

#### 2.1.3. Viral Keratitis

The most common etiological agents causing viral keratitis are *Herpes simplex virus* (HSV keratitis) and *Varicella zoster virus* (*herpes zoster* ophthalmicus) [23]. These viruses usually affect the eyelids, conjunctiva, and cornea [24].

##### Herpes Simplex Virus Keratitis

HSV type 1 (HSV-1) primarily causes infection on the face, lips, and eyes. It affects all the major ocular tissues, including the eyelids, conjunctiva, cornea, uveal tract, and retina. HSV type 2 (HSV-2) rarely infects the eye, but may occur in neonates through infected genitourinary secretions or at birth during the passage through the birth canal in the case of vaginal delivery (ophthalmia neonatorum) [25]. Herpetic infection can be classified into primary and recurrent disease. Primary infection affects the oro-facial region along with the cornea. The virus then invades the innervating trigeminal ganglia (TG), establishing a state of latency. Viral DNA is retained within neuronal nuclei without producing new infectious viral particles. Thereafter, virus reactivation results in recurrence at the primary site of infection [26]. Herpetic keratitis can have various manifestations, including the epithelial dendritic (Figure 2d) and geographic forms, which are infective. Necrotizing stromal keratitis is characterized by dense stromal infiltrate, stromal necrosis, melting, and complications such as corneal thinning, perforation, scarring, neovascularization, and lipid deposition [27]. Immune stromal keratitis and disciform keratitis are caused by the host immune reaction to the viral antigen and are a non-infective pathology (Figure 2e,f). Neurotrophic keratopathy is also a non-infective condition occurring due to impaired corneal innervation secondary to previous viral infection [25].

##### Herpes Zoster Ophthalmicus

*Varicella zoster* virus (VZV) causes chickenpox (varicella) and shingles (*Herpes zoster*). Ocular manifestation occurs by direct viral invasion, secondary inflammation, and reactivation. Hutchinson’s sign (vesicles formed at the side or tip of the nose) is indicative of higher risk of corneal involvement [23]. *Herpes zoster* ophthalmicus can include acute, chronic, and relapsing eye disease. Skin involvement with a vesicular rash affecting specific dermatomes is characteristic of this condition. Corneal, uveal, and scleral involvement can result in a painful condition and recurrent disease can cause corneal scarring and visual impairment [28,29].

#### 2.1.4. Acanthameoba Keratitis

*Acanthameoba* keratitis is caused by a free-living amoeba of the genus *Acanthamoeba.* It can cause severe sight threatening corneal ulcers. Contact lens usage is one the most common risk factors for this condition but it can also be seen with exposure to contaminated water and damage to the corneal surface. Severe pain, photophobia, and corneal infiltrate with a ringlike configuration and radial keratoneuritis may be seen [30] *(*Figure 2c,g). The diagnosis of *Acanthamoeba* keratitis can be difficult as the organism is not easily cultured in the laboratory. In vivo confocal microscopy showing classic cysts (Figure 2g) is useful but they may not always be visible. Polymerase chain reaction (PCR) and histopathological examination have also been tried with varying sensitivity [31]. The treatment includes use of topical biguanides, such as polyhexamethylene-biguanide (PHMB) and chlorhexidine in 0.02% concentration, and diamidines, such as propamidine-isethionate and dibromopropamidine in 0.1% concentration. However, results can be variable. In advanced stages therapeutic keratoplasty may be required [32].

### 2.2. Conjunctivitis

#### 2.2.1. Bacterial Conjunctivitis

Bacterial conjunctivitis primarily affects the conjunctiva. It can range from a mild self-limiting disease to severe inflammation with copious purulent discharge [10]. Acute bacterial conjunctivitis is commonly caused by *Staphylococcus aureus*, *Streptococcus pneumoniae*, and *Haemophilus influenzae*. Unilateral or bilateral redness, classically purulent discharge, photophobia, tearing, irritation, stinging, burning, and discomfort are the major symptoms of bacterial conjunctivitis [5]. Hyperacute conjunctivitis is predominantly caused by *Neisseria gonorrhoeae*, which is a common sexually transmitted pathogen [33]. Symptoms include chemosis, profuse and purulent discharge, severe eyelid edema, and tenderness and can lead to complications including peripheral and central corneal ulceration, pseudo-membrane formation, perforation, and endophthalmitis [34].

Chronic conjunctivitis is primarily caused by *Chlamydia trachomatis*, leading to trachoma [35]. Chlamydia transmission occurs through auto-inoculation from genital secretions. The infection presents as unilateral or bilateral eye redness, discharge, and watering [36]. Clinical signs include mucopurulent discharge, peripheral corneal infiltrates, large follicles on the inferior fornix and upper tarsal conjunctiva, conjunctival scarring, and tender preauricular lymphadenopathy [17]. Chlamydial infections are treated with oral tetracycline, azithromycin, and intramuscular injections of ceftriaxone [37]. This infection is usually restricted to endemic regions and is rare now. Chronic infection results in tissue destruction, scarring, corneal vascularization, cicatricial entropion with trichiasis, and corneal opacification which eventually lead to blindness [38].

#### 2.2.2. Viral Conjunctivitis

Viral conjunctivitis is a contagious acute conjunctival inflammation most commonly caused by adenovirus [39]. Symptoms comprise irritation, photophobia, and watery discharge. Dissemination of the virus occurs through fomites. *Adenovirus*, belonging to the Adenoviridae family, is a non-enveloped, double-stranded DNA virus. Adenoviral infection is regarded as the most common external contagious ocular infection globally and is usually diagnosed directly by clinical signs [40]. Viral cultures or immunodiagnostic testing are performed in few cases. Conjunctivitis may arise primarily from the viral infection of the conjunctival tissue or secondarily from ocular or systemic complications that induce conjunctival inflammation [41]. Epidemic outbreaks are categorized as the clinical syndromes of epidemic keratoconjunctivitis (EKC) which occurs in 20–40-year-old adults and pharyngeal conjunctival fever (PCF) which is more common in children [42]. Infection with a member of the Herpesvirus genus (e.g., *Herpes simplex*, *Varicella zoster*, or *Epstein–Barr virus*) can also less commonly result in acute conjunctivitis [43]. Viral conjunctivitis is a self-limiting condition which typically resolves within two weeks of the onset of symptoms. In most cases, antiviral medication is not required [44].

## 3. Diagnostic Challenges

For the successful treatment of ocular infections, it is very important to identify the causative organism. The conventional diagnostic methods used include direct microscopic visualization of the clinical sample by making a smear of the infective material on a glass slide. Specific stains such as Gram’s stain for bacteria, potassium hydroxide preparation for fungi, and additional staining such as Giemsa, Ziehl–Neelsen and lactophenol cotton blue help identify the organisms under the microscope [45]. The sample collected from the corneal scraping is also sent for culture which is the gold standard for diagnosis and it is recommended to be performed in all cases of microbial keratitis. Corneal biopsy can be collected from deeper infiltrates that are not amenable to superficial corneal scraping [46].

Microbiological procedures help make a definitive diagnosis especially in cases where there is a lack of pathognomonic clinical signs [47,48]. Blood and chocolate agar plates are the most commonly used culture media for bacteria while Sabouraud’s dextrose agar is the culture medium of choice for fungi. In vivo confocal microscopy is an additional diagnostic technique in cases of fungal and *Acanthamoeba* keratitis, where the classic appearance of the microorganism’s aids in diagnosis. However, there are situations where microbiological tests fail to give a positive result, especially in non-healing ulcers already treated with multiple medications, deep infections where it is difficult to obtain an adequate sample for testing, and mixed infections with more than one causative organism involved. Certain fastidious organisms are also difficult to culture by routine techniques. Conditions such as *Acanthamoeba* keratitis, VZV keratitis, and HSV keratitis may sometimes pose clinical dilemmas [49,50,51,52].

HSV keratitis can usually be diagnosed based on clinical features, however, in chronic conditions it is advisable to confirm the diagnosis by PCR testing. PCR is considered to have improved sensitivity for the diagnosis of HSV keratitis compared to cell culture techniques [53]. In cases where the diagnosis is elusive, using patient tears was suggested as an alternative method in determining viral load [54]. With the advent of high-throughput techniques, it may be possible to overcome the sensitivity-related concerns for tear samples. Additionally, it has been reported that several factors may decrease the PCR sensitivity in atypical lesions or lesions in patients who were previously treated with or are currently being treated with antiviral medications [55]. PCR is typically negative in stromal keratitis because the majority of the stromal infiltrates are caused by the immune response to the virus rather than active viral replication [56]. The standard culture method may be time-consuming and takes a week or longer to provide the results.

## 4. Clinical Need

The interplay between the microbial virulence and host stress response plays a crucial role in both the anatomical and functional outcomes of ocular surface infections. An ideal anatomical outcome of ocular surface infection is complete resolution of the microbial infection with minimal host tissue destruction and scarring. However, this may not always be possible and morbidity due to loss of vision remains a major ocular problem worldwide. Failure to control the infection through medical management may necessitate surgical intervention. Transplantation of the infected host corneal tissue with a healthy donor cornea can remove the infective focus but recurrence of infection can occur from the residual corneoscleral rim and the adjacent ocular tissues. At times, post-surgical recurrence can be even more challenging than treating the primary infection.

Even with successful control of microbial infection, the host ocular surface (cornea and conjunctiva) wound healing response can lead to suboptimal visual outcomes. The corneal stromal scarring, a result of host wound healing response to the microbial invasion, affects the visual acuity of the patient especially if the ulcer was large and the residual scar is in the central visual axis. Similarly, both acute and chronic conjunctival infections can lead to cicatricial sequelae including subepithelial fibrosis, symblepharon formation, and dry eye. Hence, carefully titrated approaches aimed at modulating host wound healing response through understanding the molecular microbiologic and healing events during microbial infection may help achieve optimal outcomes compared to the conventional microbicidal approaches. For such a titrated treatment approach, better diagnostic monitoring tests need to be available, which can detect the host response to the infection and treatment. Targeted medical therapy armed with the knowledge on molecular perspectives of the virulence and antimicrobial sensitivity of the microbial pathogen can help in successful medical management. To that end, it is important to have a cohesive understanding of the host molecular response to various types of ocular infections. While much remains to be learned, efforts from laboratories worldwide have helped identify the molecular profiles in patients and disease models which can lead to the development of more efficient treatment modalities as well as diagnostic or monitoring modalities.

## 5. Molecular Status of Ocular Surface Infection Mediated Host Molecular Responses

Upon infection, the ocular surface recognizes pathogens as foreign and eliminates them to maintain corneal transparency. The first line of defense includes a combination of mechanical and immunological factors which have evolved to protect the eye. We discuss the roles of various pathogen mediated host molecular responses which may contribute to prevention of eye infection. The emergence of newer high-throughput techniques has revolutionized our ability to evaluate host protein responses on a global scale, facilitating the discovery of previously unexplored mechanisms [57].

### 5.1. Bacterial Keratitis

Proteomic technologies unraveled the mechanisms of bacterium–host interaction and improved understanding of the pathogenesis of bacterial keratitis. A proteomic study on an infection model for *S. aureus* in New Zealand white rabbits by Callegan et al. revealed α-toxin as the major virulence factor in keratitis as compared to β- and γ-toxins. The α-toxins cause host ocular damage by destroying the corneal epithelium while β-toxins were found to mediate keratitis and edema in the sclera as well as conjunctiva in this study [58]. The proteomic analysis of keratitis caused by *P. aeruginosa* revealed multiple virulence factors such as elastase B (LasB), alkaline protease, exoenzyme S, slime polysaccharide, exotoxin A, endotoxin, leukocidin, phospholipase C, *P. aeruginosa* small protease (PASP), and protease IV along with cellular structures, such as pili and flagella [59]. SDS-PAGE and Western blot analyses were carried out to purify PASP and LasB and understand their role in bacterial keratitis [60]. The role of exotoxins (Exo S, Exo T, and Exo U) in *P. aeruginosa* keratitis was characterized by Western blot [60]. Exo S toxins are responsible for invasive infections and Exo U toxin leads to acute cytotoxicity in the host cells [61]. The host response against these microbial virulence factors in a protein array experiment revealed increased expression of IL-8, IL-6, and GRO in infected immortalized cell lines which showed an antibacterial effect [61]. Epithelial derived GRO primarily contributes to the recruitment of polymorphonuclear (PMS) leukocytes and secondarily it induces the corneal inflammation [62]. Global proteomic analysis by LC-MS/MS revealed 133 differentially expressed host proteins in both clinical (*P. aeruginosa*) and laboratory (*P. aeruginosa* ATCC 10145) infected strains and control samples. The upregulated proteins from the infected samples were related to pathogenicity and virulence. Two non-ribosomal peptide synthetases (NRPSs) were only present in the keratitis sample, which produced the secondary metabolite L-2-Amino-4-methoxy-trans-3-butenoic acid (AMB), regarded as a potent toxin secreted by *P. aeruginosa* [61,63]. The role of the nucleotide oligomerization domain (NOD)-like receptor (NLR) family with caspase activation and recruitment domain (CARD) containing 3 (NLRC3) was investigated in C57BL/6J mice after *P. aeruginosa* infection. Decreased levels of proinflammatory cytokines and activation of the NF-κB signaling pathway were observed when NLRC3 was overexpressed and reduced *P. aeruginosa* induced keratitis progression. The anti-inflammatory role of NLRC3 in *P. aeruginosa* induced keratitis suggested NLRC3 as a potential therapeutic target for PA induced keratitis [64]. Apart from the inflammatory proteins, the innate immune system of the host also plays an important role in protection from microbial pathogens. In the cornea, macrophages and dendritic cells play an important role in initiating the innate immune response. Recently, Hazlett et al. showed improved disease outcome after downregulation of one promising target, high mobility group box 1 (HMGB1), which promotes dendritic cell maturation, contributing to tissue pathogenesis and inflammation using small interfering RNA (siRNA) [65]. Activation of PRRs results in production of a cascade of inflammatory cytokines such as IL-1, IL-6, and IL-8 via NF-κB [66]. A myeloid differentiation primary response gene 88 (MyD88) mediated proinflammatory pathway is initiated once TLR4 and TLR5 on macrophages recognize the flagellin and lipopolysaccharide (LPS) of *P. aeruginosa* [67]. Pretreatment with flagellin has been shown to suppress early mucosal immune responses in mouse models of keratitis infection and leads to increased disease severity [68]. It has been reported that recruitment and persistence of PMN and other white blood cells are associated with corneal scarring [69]. The proteins and chemical compounds present in tear fluid, including iron, lactoferrin, peptidoglycan, phospholipase A2, defensins, and arachidonic acid metabolites [70], are associated with infections. The anti-inflammatory role of thrombomodulin in *P. aeruginosa* bacterial keratitis was explored and it was demonstrated that treatment with recombinant TM (rTM) results in protection against keratitis in B6 mice [70]. Increased expression of AnxA1 and fpr2, mediators in homeostasis of inflammation and ocular infections in infected mice, was observed and further investigations on the use of AnxA1 as a possible co-adjuvant therapeutic strategy in bacterial keratitis were suggested [71].

Significant alteration of gene expression in infected ocular surface tissues suggested that gene expression patterns and profiles are highly species specific. Studies have reported differentially expressed genes (DEGs) to distinguish bacterial and fungal keratitis. The Gene Expression Omnibus was used to download the expression profile of normal corneas and bacterial and fungal infection [72]. Amongst 451 DEGs found in bacterial keratitis, 148 DEGs were solely responsible for bacterial keratitis and 117 DEGs were co-expressed gene pairs in both fungal and bacterial keratitis. Three hundred and fifty-three specific DEGs were screened in fungal keratitis, among which 50 DEGs were strictly found in fungal keratitis and 87 DEGs were co-expressed gene pairs in both fungal and bacterial keratitis. In the fusional co-expression network by analyzing DEGs, nine biological pathways and seven KEGG pathways were revealed. Results from the network analyses indicated *SOD2* DEG as the indicator for fungal keratitis and that DEG in TLR represented bacterial keratitis, representing genes for differential analysis. Although *SOD2* does not have any descriptive role, it was shown to activate response to wounding and oxidation–reduction pathways [72]. A retrospective study was performed to evaluate contact lens keratitis by assessing the role of SNPs in *IL-10* and *IL-17* genes [73]. Buccal swab samples were collected from 88 keratitis patients, amongst them 25 were severe and 185 were healthy contact lens users, to carry out DNA extraction and SNP genotyping by pyrosequencing for *IL-10* and *IL-17* [73]. However, the SNPs did not show any relation to the severity of contact lens keratitis. SNPs in the minor allele G associated with *IL-17* showed escalated risk of severe microbial keratitis. However, this did not conclude that severity of microbial keratitis is completely dependent on genetic variation in *IL-17*, although the *IL-17* pathway is thought to be clinically important in the mechanism of microbial keratitis [73]. Microarray transcriptomic profiling of genes in bacterial keratitis and fungal keratitis patients revealed 185 unique differentially expressed genes in bacterial keratitis, 50 in fungal keratitis, and 339 common to both. In this study, MMP9, along with other MMPs (MMP1, MMP7, MMP10, MMP12), pro-inflammatory cytokines (IL1B, TNF), and PRRs (TLR2, TLR4), were upregulated in bacterial and fungal keratitis. HIF1A and its induced genes were upregulated uniquely in bacterial keratitis [74]. Gowda et al. found constitutively high expression of Pglyrp-1 in the superficial cells of the corneal epithelium in mouse and human corneas infected with *P. aeruginosa* which suggested the protective role of Pglyrp-1 at the ocular surface. They showed that *Pglyrp-1-/-* mice challenged with *P. aeruginosa* keratitis showed poor bacterial clearance and resolution of keratitis [75], while disease severity was reduced with improved bacterial clearance in *Pglyrp-2-/-* mice that may be due to compensatory overexpression of defensins (mBD-2 and mBD-3), cathelicidin-related antimicrobial peptides (Cnlp), and Pglyrp-1 [75] (Table 2 and Table 3).

### 5.2. Fungal Keratitis

Fungal keratitis (FK) is regarded as one of the most severe corneal infections. A tear proteome delivered comprehensive details regarding host ocular surface protein profile related to tissue injury and defense responses during FK. Differential expression of representative host response proteins causing FK can be implemented as biomarkers to establish the clinical prognosis and titrate the treatment and management strategy in distinct stages of FK [76]. The tear proteome of FK showed expression of a glutaredoxin-related (GRX) protein, which was secreted by *Aspergillus* sp. under oxidative stress. GRX is known to be involved in several cellular activities, i.e., protein folding, sulfur metabolism, protection of cells from oxidative stress, and DNA synthesis. Amongst the six abundant tear proteins released as host response, prolactin inducible protein and serum albumin were upregulated in the FK group. On the other hand, expressions of cystatin SN precursor, cystatin S precursor, lipocalin, and cystatin were downregulated [77]. Ananthi et al. further performed proteomic analysis in *Fusarium* keratitis infected eyes and control healthy subjects’ tears from different clinical stages. The tear groups of normal subjects, and early, intermediate, and late clinical stages *Fusarium* keratitis were pooled and analyzed. The authors performed two-dimensional difference gel electrophoresis (2D-DIGE) to assess the low-abundance proteins and improve protein separation. Liquid chromatography–tandem mass spectrometry (LC-MS/MS) was conducted for further protein identification and segmentation [78]. Different stages of *Fusarium* keratitis showed different expressions of host response proteins. As the disease progressed towards the late stages of FK, several proteins, i.e., α-1-antitrypsin, zinc-α-2-glycoprotein, haptoglobin α2 chain, albumin, apolipoprotein, lactoferrin, and haptoglobin precursor-β-chain were gradually upregulated. α-1-antitrypsin inactivates the microbial enzymes which act as an acute level reactant. Apolipoproteins are responsible for metabolism and uptake regulation of lipoproteins. The expression of lacritin precursor was downregulated to a negligible level in the early stage, compared to the control. During the later stage of *Fusarium* keratitis, the level of cystatin SA III and lipocalin was decreased [78]. Kandhavelu et al. segregated tear proteins by 1D-PAGE, glycosylation, and in-gel digestion to identify host response proteins by LC-MS/MS. The tear protein profiles of pooled tears from early *Aspergillus* keratitis and normal subjects were compared. The presence of proteins specific for neutrophil extracellular traps, proteins involved in wound healing, and complement system proteins was found only in the FK tears. Identification of host defense proteins and wound healing proteins at the early stages of *Aspergillus* keratitis may help in tracking *Aspergillus* keratitis progression [79]. Calvillo-Medina et al. analyzed the ability to form biofilms in vitro by *F. falciforme* isolated from FK corneal scrapes and examined its protein expression. They conducted 2D-PAGE separation for protein identification by MALDI-TOF. They found that 19 proteins were upregulated in biofilms, and amongst them six proteins showed unique expression. Relatively abundant proteins included enolase, ATP-citrate synthase, phosphoglycerate kinase, and transketolase. Some of these proteins were found to be associated with basal metabolism and act as potential virulence factors [80]. Mixed infection in microbial keratitis may occur from antibiosis of bacterial and fungal pathogens. As a result, proteomics analysis might explore the biology behind the mixed biofilm formation caused by bacteria and fungi.

*Aspergillus flavus* and *Fusarium solani* predominantly cause corneal mycotic ulceration in tropical countries [81]. Tear proteome profiles of *Aspergillus keratitic* patients were examined at different stages of infection. Profiling of the proteome was performed by 2D-PAGE and 2D-DIGE was carried out to quantify the protein levels. Upregulation of apolipoprotein, alpha-1-antitrypsin, lactoferrin, haptoglobin, and albumin was observed in the tear fluid of patients [82]. However, expressions of lacrimal lipocalin precursor, cystatin SA III precursor, zinc alpha-2 glycoprotein (ZAG), and lacritin precursor were downregulated. As the disease progressed from early to late stages, all the proteoforms of ZAG were concomitantly downregulated. There was no difference in the ZAG expression level in the keratitic tear film according to gender. Early events of host response showed upregulation of ZAG in Fusarium keratitis infection, highlighting its potential as a diagnostic biomarker. ZAG breaks down lipid in adipocytes but the exact role of ZAG in tears has not been studied intensively [82]. Biofilm-forming capability and antibiotic susceptibility of the ocular isolates of *Candida albicans* were studied and gene expression has been reported [83] in six keratitis and one orbital cellulitis clinical isolate. Biofilm formation was monitored by scanning electron microscopy (SEM) and confocal laser scanning microscopy (CLSM). Potential biofilm formation was observed in four ocular isolates along with resistance to three antifungal medications also described in one isolate. The rest of the isolates were susceptible to all the antifungal medications [83]. Two to three adherent layers of cells present at 24 h increased to multiple layers in 72 h according to the SEM studies. CLSM revealed that biofilm thickness increased to 17.98 μm at 72 h from 5.2 μm at 24 h [83]. Biofilm positive ocular and non-ocular *C. albicans* isolates showed upregulation of 27 genes, whose expression was similar in both non-ocular pathogenic *C. albicans* and biofilm-forming ocular isolates. These 27 genes were involved in the adhesion, initiation, maturation, and dispersal stages of biofilm. The expression pattern followed four different patterns of the biofilm-forming stages of the temporal expression in biofilm-positive ocular isolates. The similarity in gene expression between biofilm-forming ocular and non-ocular *C. albicans* isolates indicated that upregulated genes can be used as a possible therapeutic target. Transcriptome analysis of fungal keratitis revealed inflammatory cytokine genes, i.e., IL-1B, IL-6, TNF- α, to be significantly associated with fungal keratitis. Pathway enrichment analysis showed Wnt, cGMP–PKG, and Hippo signaling pathways to be responsible for the pathogenesis of fungal keratitis [84]. At the advanced stages of fungal keratitis, the levels of IL-1β, IL-6, IL-8, and IFN-γ in the aqueous humor were shown to be significantly increased [85]. Along with the cytokine expression, significantly higher expression of macrophage inducible Ca2+-dependent lectin receptor (Mincle) was observed during the early period of *Aspergillus fumigatus* infection in rats, which may play a role in the early host innate immune response of the corneal resistance against fungus [86] (Table 2 and Table 3).

### 5.3. Viral Infections

Proteomic profiling of corneal epithelial cells infected with HSV-1 was performed. Network analysis revealed the protein groups involved in mRNA splicing, ATP synthesis and post-translational protein folding, RNA processing, and gene expression [87,88].

Herpetic stromal keratitis (HSK) is caused by infection by *Herpes Simplex* virus (HSV) in the cornea. The interaction between cornea infiltrating inflammatory cells and resident cells in macrophages produces IL-1, TNF-α, and IFN-γ which in turn generate Th1 cells [89]. Corneal lesions and blindness are caused by a major influx of neutrophils and sustained local secretion of immune modulatory factors. IL-17 plays an important role in the massive infiltration of neutrophils into inflamed tissues [90]. Human corneal fibroblast (HCF) expresses IL-17R constitutively and HSK tissues express IL-17. Induction of IL-6 and IL-8 secretion by cultured HCF resulted in a synergistic effect between IL-17 and TNF- [90]. A strong chemotactic effect was observed in the neutrophils due to the secretion of IL-8 by HCF. IL-17 inhibited the secretion of RANTES; on the other hand, it induced the secretion of macrophage inflammatory proteins (MIPs) 1α and 3α. IFN-γ-related protein, i.e., IP10 and matrix metalloproteinase 1, levels were considerably elevated and the monocyte chemotactic protein 1 level remained unaltered. These data suggested that IL-17 might be an important factor in modulating the proinflammatory and neutrophil chemotactic factors in the corneal resident fibroblasts, resulting in the enhancement of the immunopathologic processes in human HSK [90]. The HSV-1 latency reactivation cycle gives rise to significant human pathology [91]. The HSV-1 latency associated transcript (LAT) regulates latency and reactivation by inhibiting apoptosis [92]. The *Herpes Simplex virus* (HSV-1) latency associated transcript (LAT) is associated with inhibiting apoptosis via hindering the activation of proapoptotic caspases. It was observed that LAT inhibited apoptosis by regulating the expression of apoptotic genes [92]. The molecular mechanism of antiapoptotic functions of LAT at a transcriptional level suggests that (i) LAT probably impedes apoptosis via upregulation of different components of the type I interferon (IFN) pathway; (ii) inhibition of apoptosis by LAT is neither accompanied by downregulation of Toll-like receptor (TLR) nor via caspase cascade at a transcriptional level. These factors suggested that immune exhaustion was not brought about by the antiapoptotic activity of the LAT [92]. miR-155 played a dominant role in HSK by regulating the immune system. HSV-1 infection of mouse cornea resulted in enhanced upregulation of miR-155 at 2, 7, and 15 days post-infection [93]. This upregulation was observed especially in activated CD4+ T cells, along with neutrophils in the infected cornea. On the contrary, the severity of the infection is reduced and accompanied by reduced angiogenesis and infiltration of CD4+ T cells, diminished Th1 and Th17 response in the infected cornea, and draining lymph nodes (DLNs) and lymphoid organs in miR-155 knockout mice [94]. The decreased proliferation of CD4+ T cells results in a decreased number of infiltrating CD4+ T cells, suggesting the role of miR-155 in promoting CD4+ T cell proliferation [94]. The in vivo silencing of miR-155 by injecting antigomir-155 nanoparticles in the conjunctiva diminished the severity of HSK with less infiltration of CD4+ T cells and neutrophils along with decreased production of proinflammatory cytokines, including IL-1, IL-6, IL-17, and IFN and chemokines, e.g., Ccl-2 and Cxcl-1 [94]. The antiangiogenic effect of antigomiR-132 accompanied an enhanced p120RasGAP expression and Ras activity was minimized in the endothelial cells of the cornea [95]. This concluded that p120RasGAP brings about the pro neovascularization function of miR-1320s, suggesting the knockdown of miR-132 can be a potential therapeutic alternative for HSK treatment [95]. The molecular basis of ocular HSV-1 infection has led to the identification of inhibitors of TANK-binding kinase 1 (TBK1) such as BX795, which strongly suppressed infection by multiple strains of HSV-1 in vivo [96]. The antiviral activity of BX795 targeted Akt phosphorylation in infected cells, resulting in the blockage of viral protein synthesis. It established the fact that BX795 can be used as a promising alternative broad-spectrum antiviral application in humans [96]. Recently, the effect of RNA interference in HSV keratitis was studied for prophylaxis and therapy by targeting glycoprotein D (gD) and glycoprotein E (gE). The expression of mRNA encoding gD and gE showed a decrease in the viral titer when used for prophylaxis rather than therapy. This result indicated the prophylactic role of small interfering RNA in HSV keratitis [97]. Yang et al. investigated the HSV-1 epithelial keratitis tear proteome by nano-LC/MS. Three hundred and twenty-six unique proteins were found in HSV keratitis samples. Functional annotation by gene ontology (GO) revealed most of the proteins are involved in antigen presentation, metabolic processes, and TNF mediated and T cell mediated inflammatory pathways. Levels of IL1A, IL12B, DEFB4A, and CAMP proteins were significantly higher in HSV keratitis samples, indicating higher levels of viral inhibition and inflammatory response. These unique proteins can be quantified by ELISA for the discovery of biomarkers for rapid diagnosis of HSV-1 epithelial keratitis [98]. HSK showed a significant decrease in MIP-1α deficient (−/−) hosts although virus replication and clearance did not differ significantly from that seen in infected wild-type (+/+) mice and it was concluded that MIP-1α is not needed to control virus growth in the cornea but is essential for the development of severe stromal keratitis [99]. The levels of Substance P in the infected corneas were evaluated by enzyme immunosorbent assay (EIA), which showed approximately a 15-fold higher amount of Substance P in the corneas with severe HSK lesions in comparison with those with mild HSK lesions, contributing to the clinical severity of HSK lesions in a mouse model [100]. Epithelial cells act as a critical barrier in protecting the cornea from microbial pathogen infection. The expression of TRIM32 was increased after infection with HSV-1 both in murine corneas and cultured human epithelial (HCE) cells. Furthermore, knockdown of the expression of TRIM32 significantly aggravated HSV-1 induced herpetic stromal keratitis (HSK) in mice and promoted the replication of HSV-1 in cultured HCE cells. The decreased expression of IFN-β and suppressed activation of interferon regulatory factor 3 (IRF3) positively regulated HSV-1 infection [87]. The induction of MIP-2 which in turn promoted the recruitment of neutrophils to the infected cornea was tested in a Chinese hamster model [101]. Hyaluronic acid (HA), which is an important component of the extracellular matrix, plays an important role in tissue development, cell migration, cell proliferation, and inflammation and was found to be elevated in tear fluid of patients with adenoviral conjunctivitis [102] (Table 2 and Table 3).

## 6. Translational and Clinical Relevance

**Figure 3:**
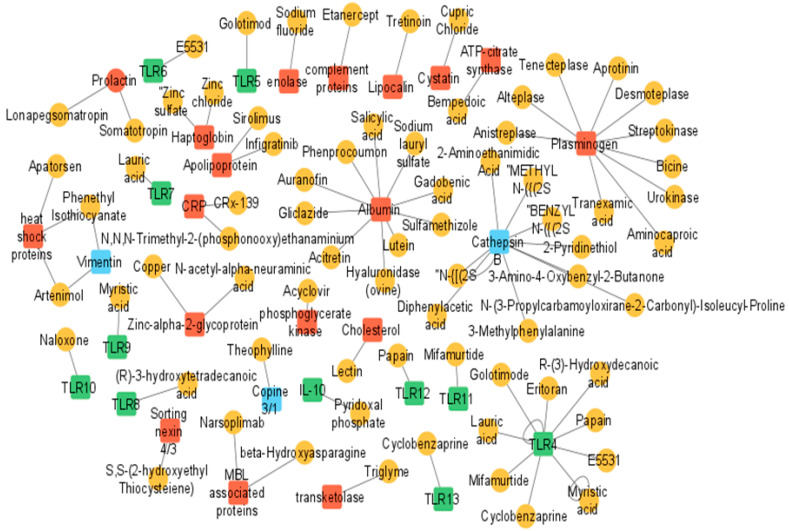
Drug–target interaction network constructed using DrugBank database. Drugs and targets are presented by circles and squares, respectively. Drug–target interactions are represented by the lines connecting related drugs and targets. Green (square) targets represent bacterial infection, blue (square) targets represents viral infection, and red (square) targets represent fungal infection.

Microbial ocular infection mediated host stress responses aid in understanding the etiology and pathogenesis of ocular infections important for early diagnosis and effective treatment. Elucidation of the mechanisms of these host stress responses can further aid the development of therapeutics, such as topical pharmacologic or molecular targets, to block or promote the production of stress mediators. Currently, bacterial ocular infections are treated using broad-spectrum antibiotics such as cephalosporins, fluoroquinolones, and aminoglycosides. Fungal and viral infections are treated by prescribing antifungal and antiviral medications such as fluconazole, clotrimazole, and oral acyclovir. These medications prevent further spread of infection in other parts of the eye, but are often very broad spectrum and are not successful in addressing all the aspects of a patient’s pathology. Apart from this, treatment directed to reduce host cell/tissue damage which also maintains the host cell homeostasis during infection could be critical to prevent further deterioration in visual acuity, which is currently an unmet clinical need. In this context, we have collated the host tissue molecular response data and searched for experimental, investigational, and approved drugs against each of the targets in the DrugBank database [103] and constructed a drug–target network. We present a network visualization of predicted drug–target associations that could provide helpful information for the discovery of new therapeutic modalities which may improve the visual acuity of patients after treatment (Figure 3).

## 7. Conclusions

It is very evident from the molecular data presented by various groups that different types of ocular surface infections lead to unique host cellular and molecular responses. The host stress response against bacterial keratitis showed an increase in proinflammatory markers. Expression of cystatin SN proteins, an endogenous proteinase inhibitor involved in lipid metabolism and host wound healing responses, was shown to be associated with host stress responses after fungal ocular infections. Modulation of host response proteins involved in intracellular trafficking, proteases, maintenance of cellular integrity, and resistance against stress showed modulations after viral ocular infections. There are very few reports on host molecular responses in microbial keratitis and no reports with respect to conjunctivitis infection. Studying the responses of host in ocular infections in further detail may enhance our knowledge of pathogenesis along with their cellular functions in host cells. Several diagnostic techniques are used in clinics to differentiate between ocular infections. The proper diagnosis of the exact causative organism is very important, which will lead to successful treatment at the preliminary stage and thus lessen the morbidity resulting from different causative organisms. The techniques that are available and largely used need a corneal scraping as a sample which is invasive and may be painful and may not give the necessary sample in the case of deep infections. Non-invasive diagnostic techniques should be developed which are not time-consuming and are definitive for the infections. High-throughput molecular techniques such as proteomics should be explored to extract the targets which can be validated as diagnostic and therapeutic readouts.

## Figures and Tables

**Table 1:** Types of ocular infections and their pathophysiology.

Ocular Infection	Causative Agent	Sign/Symptoms	Treatment
Bacterial Keratitis	*Pseudomonas aeruginosa* *Staphylococcus aureus* *Streptococcus pneumoniae*	Symptoms: Eye pain, blurred vision, photophobia, and discharge Signs: Corneal infiltrate of varying involvement of corneal stromal depth and diameter with overlying epithelial defect. Severe infections can be associated with anterior chamber inflammation and hypopyon formation. Progressive ulceration can result in complications including corneal perforation, scleritis, and endophthalmitis	Topical antibiotics are used based on microbiological sensitivity. Combination of medications may be required
Fungal Keratitis	Filamentous fungi (*Aspergillus* sp., *Fusarium* sp., *cladosporium* sp.) Yeast (*Candida* sp.)	Symptoms: Eye pain, blurred vision, photophobia, and discharge Signs: Dry looking base of corneal ulcer base with feathery margins. Can also have satellite lesions, endoexudates and hypopyon	Topical antifungal medications as per severity of infections. Natamycin and voriconazole are the most common medications used. Oral antifungals of the azole group are also given for more severe infections
Viral Keratitis	*Herpes simplex virus* *Herpes zoster Ophthalmicus*	Symptoms: Discomfort, watering, itching, burning, and pain in the eye Signs: Would depend on the form of the herpes viral involvement	Topical acyclovir and ganciclovir for epithelial forms of disease. Stromal viral keratitis requires topical steroids. Viral endothelitis and recurrent stromal involvement require oral antiviral medications
Bacterial Conjunctivitis	*Staphylococcus aureus*; *Streptococcus pneumoniae* (acute bacterial conjunctivitis) *Neisseria gonorrhoeae* (hyperacute conjunctivitis)	Symptoms: Unilateral or bilateral redness, classically purulent discharge, photophobia, tearing, irritation, stinging, burning, and discomfort Signs: Diffuse bulbar conjunctival injection. Discharge is initially watery, becomes purulent as infection progresses	Acute bacterial conjunctivitis is usually treated with topical fluoroquinolones, macrolides, and aminoglycosides. Tetracycline and macrolides are used to treat chlamydia
Trachoma	*Chlamydia trachomatis* serotypes A, B, Ba, C	Active disease: Mucopurulent discharge, superior epithelial keratitis, pannus formation, superior conjunctival follicles on upper limbus Chronic disease: Conjunctival scarring, corneal opacification, cicatricial entropion, and trichiasis	SAFE strategy: S: Surgery in case of trichiasis, A: Antibiotics (azithromycin and erythromycin) for C. trachomatis infection, F: Facial cleanliness, E: Environmental change for sanitation
Viral Conjunctivitis	*Adenovirus* *Herpes simplex virus*	Symptoms: Unilateral or bilateral watering, redness, discomfort, and photophobia Signs: Eyelid edema, follicular conjunctivitis, tender preauricular lymphadenopathy. Chronic cases can have mild conjunctival scarring	Symptomatic relief and cold compresses. Topical antibiotics to prevent secondary infection and topical steroids for severe inflammation in conjunctivitis

**Table 2:** Host response proteins in ocular microbial infections.

Ocular Disease	Techniques Used	Host Molecular Responses	References
Bacterial keratitis	SDS-PAGE, Western blot, ELISA Western blot, ELISA, protein array, and LC-MS/MS ELISA and Western blot	*Staphylococcus aureus* keratitis: α-toxin, β-toxin Upregulated soluble factors: IL-4, IL-6, and IL-10 *Pseudomonas aeruginosa* keratitis: Upregulated soluble factors in infected cornea: IL-6, IL-1β, IFN-γ, IL-12 p40, TNF-α Upregulated soluble factors in immortalized cell lines: IL-8, IL-6, and GRO. Upregulated proteins: 133 pathogenic and virulent proteins, 2 non-ribosomal peptide synthetases (NRPSs), L-2-Amino-4-methoxy-*trans*-3-butenoic acid (AMB), pneumolysin	Callegan, M. C. et al. (1994) Sewell, A. et al. (2014) Bouhenni, R. et al. (2015)
Fungal keratitis	MADI-TOF MS 2D-DIGE with LC-MS/MS 1D-PAGE with LC-MS/MS 2D-PAGE with MALDI-TOF MS 2D-DIGE Multiplex bead-based Luminex liquid protein array	*Aspergillus* keratitis: Glutaredoxin-related protein Upregulated proteins: Prolactin inducible protein and serum albumin precursor. Downregulated proteins: Cystatin S precursor, cystatin SN precursor, cystatin, and human tear lipocalin *Fusarium* keratitis: Upregulated proteins: Haptoglobin, apolipoprotein, albumin. Downregulated proteins: Lacritin *Aspergillus* keratitis: Host defense proteins: CRP, Sap, lectins, MBL associated proteins, complement proteins Wound healing proteins: thrombin, plasminogen, heat shock proteins *Fusarium falciforme* keratitis: Transketolase, a putative antigen 1, enolase, phosphoglycerate kinase, and ATP-citrate synthase. Upregulation of ZAG in the early stage. Downregulation of ZAG in the late phase of fungal keratitis. Increased expression of IL-1β, IL-6, IL-8, and IFN-γ in aqueous humor	Kandhavelu, J. et al. (2017) Ananthi, S. et al. (2008) Kandhavelu, J. et al. (2017) Calvillo-Medina, R. P. (2019) Parthiban, N. et al. (2019) Zhang, Y. et al. (2018)
Viral keratitis	iTRAQ coupled to LC–MS/MS Nano-LC/MS and ELISA Enzyme immunosorbent assay (EIA)	HSV keratitis: Upregulated proteins: Beta-globin, cathepsin B, vimentin, copine 3 Downregulated proteins: Sorting nexin 4, neurolysin, syntaxin 12 Upregulated proteins: IL1A, IL12B, DEFB4A, and CAMP 15-fold higher expression of Substance P	Berard, A. R. et al. (2015) Yang, H. et al. (2020) Twardy, B.S. et al Brandon, S. (2011)
Viral conjunctivitis	ELISA	Elevated levels of hyaluronic acid (HA) can acts as a rapid diagnostic marker	Dreyfuss, J.L. et al Juliana (2015)

**Table 3:** Host response genes in ocular microbial infections.

Ocular Infection	Techniques	Host Molecular Responses	References
Viral Keratitis	RNA isolation and real-time PCR PCR, TaqMan RT-PCR, qRT-PCR MicroRNA expression by Northern blot SiRNA expression targeting glycoprotein D and E Knockdown of TRIM32 expression in HCE HSV injection and preparation of MIP-1α deficient (−/−) mice and their wild-type (+/+)	IL-17 HSV-1 latency associated transcript (LAT) miR-155 Prophylactic role of small interfering RNA Upregulation of TRIM32 aggravates HSV keratitis by increased HSV-1 replication MIP-1α deficient mice show decreased HSK infection, plays a positive role in development of HSV keratitis	Maertzdorf, J. et al. (2002) Tormanen, K. et al. (2019) Banerjee, A. et al. (2010) Chen, L. et al. (2021) Cui, H. et al. (2017) Terrence, M. T. et al. (1998)
*Ebola virus* uveitis	RNA sequencing, RT-qPCR	Type I interferon (IFN)	Smith, J. R. et al. (2017)
Bacterial keratitis	Comparative analysis of differentially expressed genes (DEGs) by microarray data. Genotyping performed by pyrosequencing RT-PCR Pglyrp-1(-/-) mice Microarray transcriptomic profiling RT-PCR RT-PCR RNA isolation and qPCR Silencing HMGB1 by siRNA	*TLR4* and *SOD2* SNPs in minor allele G associated with *IL-17* High expression of *Pglyrp-1*. Shows a protective role of *Pglyrp-1* at the ocular surface Overexpression of defensins (mBD2 and 3), cathelicidin-related antimicrobial peptides (Cnlp) and Pglyrp-1. Upregulated cytokines in fungal and bacterial keratitis: MMPs (MMP1, MMP7, MMP10, MMP12), proinflammatory cytokines (IL1B, TNF), and PRRs (TLR2, TLR4), Increased expression of HIF1 gene in bacterial keratitis Increased expression of AnxA1 and fpr2, mediators in homeostasis of inflammation Anti-inflammatory role of thrombomodulin suggests recombinant TM (rTM) results in protection against keratitis Overexpression of NLRC3 attenuated *P. aeruginosa* induced keratitis progression, inhibited the activation of the NF-κB signaling pathway. NLRC3 can act as a potential therapeutic target for PA induced keratitis Downregulation of high mobility group box 1 (HMGB1) improves bacterial keratitis	Tian, R. et al. (2020) Carnt, N. A. et al. (2019) Gowda, R.N. et alRanjita et al. (2015) Chidambaram, J. et al. (2017) Boyd, K. et alDa Silva (2019) Sharon, A. et al. (2015) Guo, L. et al. (2017) Hazlett, L. D. et al. (2016)
Fungal keratitis	Real-time PCR Transcriptome analysis by RNA sequencing, validation with qRT-PCR RT-PCR	27 genes involved in the adhesion, initiation, maturation, and dispersal stages of biofilm. Markers of FK: Inflammatory cytokine genes: IL-1B, IL-6, TNF-α, Enriched pathways: Wnt, cGMP–PKG, and Hippo signaling pathways Higher expression of Mincle, which helps in corneal resistance and host immune response	Ranjith, K. et al. (2018) Zhang, Q. et al. (2020) Zhao, G. et al. (2015)
*Acanthameoba keratitis*	MIP-2 and myeloperoxidase (MPO) assays	MIP-2 induces neutrophil infiltration which acts as a therapeutic strategy in *Acanthameoba keratitis*	Hurt, M. et al. (2001)

## References

[1] Cher I. (2013). Ocular surface concepts: Development and citation. Ocul. Surf..

[2] Srinivasan M. (2007). Infective keratitis: A challenge to Indian ophthalmologists. Indian J. Ophthalmol..

[3] Marquart M.E., O’Callaghan R.J. (2013). Infectious keratitis: Secreted bacterial proteins that mediate corneal damage. J. Ophthalmol..

[4] Sridhar M.S. (2018). Anatomy of cornea and ocular surface. Indian J. Ophthalmol..

[5] Garfunkel L.C., Kaczorowski J., Christy C. (2007). Pediatric Clinical Advisor E-book: Instant Diagnosis and Treatment.

[6] St. Leger A.J., Caspi R.R. (2018). Visions of eye commensals: The known and the unknown about how the microbiome affects eye disease. Bioessays.

[7] Amescua G., Miller D., Alfonso E. (2012). What is causing the corneal ulcer? Management strategies for unresponsive corneal ulceration. Eye.

[8] Shah A., Sachdev A., Coggon D., Hossain P. (2011). Geographic variations in microbial keratitis: An analysis of the peer-reviewed literature. Br. J. Ophthalmol..

[9] Garg P., Rao G.N. (1999). Corneal ulcer: Diagnosis and management. Community Eye Health.

[10] Azari A.A., Barney N.P. (2013). Conjunctivitis: A systematic review of diagnosis and treatment. JAMA.

[11] Prajna V.N., Nirmalan P.K., Saravanan S., Srinivasan M. (2007). Economic analysis of corneal ulcers in South India. Cornea.

[12] Go E.P., Wikoff W.R., Shen Z., O’Maille G., Morita H., Conrads T.P., Nordstrom A., Trauger S.A., Uritboonthai W., Lucas D.A. (2006). Mass spectrometry reveals specific and global molecular transformations during viral infection. J. Proteome Res..

[13] Kosch R., Delarocque J., Claus P., Stefanie C., Jung B.K. (2018). Gene expression profiles in neurological tissues during West Nile virus infection: A critical meta-analysis. BMC Genom..

[14] Mejias A., Dimo B., Suarez N.M., Garcia C., Suarez-Arrabal M.C., Jartti T., Blankenship D., Jordan-Villegas A., Ardura M.I., Xu Z. (2013). Whole blood gene expression profiles to assess pathogenesis and disease severity in infants with respiratory syncytial virus infection. PLoS Med..

[15] Rahman Z.A., Harun A., Hasan H., Mohamed Z., Noor S.S., Deris Z.Z., Ismail N., Hassan A.S., Ahmad F., Yaakub A. (2013). Ocular surface infections in northeastern state of malaysia: A 10-year review of bacterial isolates and antimicrobial susceptibility. Eye Contact Lens.

[16] Kaufman H.E., Barron B.A., McDonald M.B. (1998). The Cornea, on CD-ROM.

[17] Kanski J.J., Bowling B. (2007). Clinical Ophthalmology. A Systematic Approach.

[18] O’brien T. (2003). Management of bacterial keratitis: Beyond exorcism towards consideration of organism and host factors. Eye.

[19] Song A., Deshmukh R., Lin H., Ang M., Mehta J.S., Chodosh J., Said D.G., Dua H.S., Ting D. (2021). Post-keratoplasty infectious keratitis: Epidemiology, risk factors, management, and outcomes. Front. Med..

[20] Dikmetaş Ö., Deniz Y., Kocabeyoğlu S., Başol M., İrkeç M. (2020). The Value of Fortified Aminoglycoside/Cephalosporin Treatment as First-Line Treatment and in Fluoroquinolone-Resistant Bacterial Keratitis. Turk. J. Ophthalmol..

[21] Niu L., Liu X., Ma Z., Yin Y., Sun L., Yang L., Zheng Y. (2020). Fungal keratitis: Pathogenesis, diagnosis and prevention. Microb. Pathog..

[22] O’day D.M., Head W.S., Robinson R.D., Clanton J.A. (1986). Corneal penetration of topical amphotericin B and natamycin. Curr. Eye Res..

[23] Abbouda A., Abicca I., Alio J. (2016). Infectious keratitis following corneal crosslinking: A systematic review of reported cases: Management, visual outcome, and treatment proposed. Seminars in Ophthalmology.

[24] Valerio G.S., Lin C.C. (2019). Ocular manifestations of herpes simplex virus. Curr. Opin. Ophthalmol..

[25] Farooq A.V., Shah A., Shukla D. (2010). The role of herpesviruses in ocular infections. Virus Adapt. Treat..

[26] Kwon M.S., Carnt N.A., Truong N.R., Pattamatta U., White A.J., Samarawickrama C., Cunningham A.L. (2018). Dendritic cells in the cornea during Herpes simplex viral infection and inflammation. Surv. Ophthalmol..

[27] Ahmad B., Patel B.C. (2019). Herpes simplex keratitis. Prog. Retin. Eye Res..

[28] Liesegang T.J. (2001). Herpes simplex virus epidemiology and ocular importance. Cornea.

[29] Rowe A., St Leger A.J., Jeon S., Dhaliwal D.K., Knickelbein J.E., Hendricks R.L. (2013). Herpes keratitis. Prog. Retin. Eye Res..

[30] Szentmáry N., Daas L., Shi L., Laurik K.L., Lepper S., Milioti G., Seitz B. (2019). Acanthamoeba keratitis–Clinical signs, differential diagnosis and treatment. J. Curr. Ophthalmol..

[31] Lorenzo-Morales J., Khan N.A., Walochnik J. (2015). An update on Acanthamoeba keratitis: Diagnosis, pathogenesis and treatment. Parasite.

[32] Lindquist T.D. (1998). Treatment of Acanthamoeba keratitis. Cornea.

[33] American Optometric Association (2010). Care of the Patient with Hyperopia.

[34] Rietveld R.P., Riet G., Bindels P.J., Sloos J.H., van Weert H.C. (2004). Predicting bacterial cause in infectious conjunctivitis: Cohort study on informativeness of combinations of signs and symptoms. BMJ.

[35] Satpathy G., Behera H.S., Ahmed N.H. (2017). Chlamydial eye infections: Current perspectives. Indian J. Ophthalmol..

[36] Deschênes J., Seamone C., Baines M. (1990). The ocular manifestations of sexually transmitted diseases. Canadian journal of ophthalmology. J. Can. D’ophtalmol..

[37] Sheikh A., Hurwitz B., van Schayck C.P., McLean S., Nurmatov U. (2012). Antibiotics versus placebo for acute bacterial conjunctivitis. Cochrane Database Syst. Rev..

[38] Hu V.H., Holland M.J., Burton M.J. (2013). Trachoma: Protective and pathogenic ocular immune responses to Chlamydia trachomatis. PLoS Negl. Trop. Dis..

[39] Sow A., Kane H., Ka A.M., Hanne F.T., Ndiaye J.M.M., Diagne J.P., Nguer M., Sow S., Saheli Y., Sy E.H.M. (2017). Senegalese experience with acute viral conjunctivitis. J. Fr. D’ophtalmol..

[40] Haq A., Wardak H., Kraskian N. (2013). Infective conjunctivitis–its pathogenesis, management and complications. Common Eye Infections.

[41] Hierholzer J.C., Wigand R., Anderson L.J., Adrian T., Gold J.W. (1988). Adenoviruses from patients with AIDS: A plethora of serotypes and a description of five new serotypes of subgenus D (types 43–47). J. Infect. Dis..

[42] Solano D., Fu L., Czyz C.N. (2017). Viral Conjunctivitis.

[43] Gordon J. (1994). Adenovirus and other non-herpetic viral diseases. The Cornea.

[44] McLeod S.D. (1997). The role of cultures in the management of ulcerative keratitis. Cornea.

[45] Sharma S. (2012). Diagnosis of infectious diseases of the eye. Eye.

[46] McLeod S.D., Kolahdouz-Isfahani A., Rostamian K., Flowers C.W., Lee P.P., McDonnell P.J. (1996). The role of smears, cultures, and antibiotic sensitivity testing in the management of suspected infectious keratitis. Ophthalmology.

[47] Kim E., Chidambaram J.D., Srinivasan M., Lalitha P., Wee D., Lietman T.M., Whitcher J.P., Van Gelder R.N. (2008). Prospective comparison of microbial culture and polymerase chain reaction in the diagnosis of corneal ulcer. Am. J. Ophthalmol..

[48] Yoder J.S., Verani J., Heidman N., Hoppe-Bauer J., Alfonso E.C., Miller D., Jones D.B., Bruckner D., Langston R., Jeng B.H. (2012). Acanthamoeba keratitis: The persistence of cases following a multistate outbreak. Ophthalmic Epidemiol..

[49] Miserocchi E., Fogliato G., Bianchi I., Bandello F., Modorati G. (2014). Clinical features of ocular herpetic infection in an Italian referral center. Cornea.

[50] Baratz K.H. (2012). The role of antiviral therapy after the resolution of acute herpes simplex keratitis or acute herpes zoster ophthalmicus. Arch. Ophthalmol..

[51] Liesegang T.J. (2004). Herpes zoster virus infection. Curr. Opin. Ophthalmol..

[52] Edell A.R., Cohen E.J. (2013). Herpes simplex and herpes zoster eye disease: Presentation and management at a city hospital for the underserved in the United States. Eye Contact Lens.

[53] El-Aal A.M.A., El Sayed M., Mohammed E., Ahmed M., Fathy M. (2006). Evaluation of herpes simplex detection in corneal scrapings by three molecular methods. Curr. Microbiol..

[54] Satpathy G., Mishra A.K., Tandon R., Sharma M.K., Sharma A., Nayak N., Titiyal J.S., Sharma N. (2011). Evaluation of tear samples for Herpes Simplex Virus 1 (HSV) detection in suspected cases of viral keratitis using PCR assay and conventional laboratory diagnostic tools. Br. J. Ophthalmol..

[55] Kowalski R.P., Gordon Y.J., Romanowski E.G., Araullo-Cruz T., Kinchington P.R. (1993). A comparison of enzyme immunoassay and polymerase chain reaction with the clinical examination for diagnosing ocular herpetic disease. Ophthalmology.

[56] McGilligan V., Moore J.E., Tallouzi M., Atkinson S.D., Neill H.O., Feeney S., Novitskaya E.S., Sharma A., Shah S., Jackson J.A. (2014). A comparison of the clinical and molecular diagnosis of herpes simplex keratitis. Open J. Ophthalmol..

[57] Bartee E., McCormack A., Früh K. (2006). Quantitative membrane proteomics reveals new cellular targets of viral immune modulators. PLoS Pathog..

[58] Callegan M.C., Engel L.S., Hill J.M., O’Callaghan R.J. (1994). Corneal virulence of Staphylococcus aureus: Roles of alpha-toxin and protein A in pathogenesis. Infect. Immun..

[59] Strateva T., Mitov I. (2011). Contribution of an arsenal of virulence factors to pathogenesis of Pseudomonas aeruginosa infections. Ann. Microbiol..

[60] Sewell A., Dunmire J., Wehmann M., Rowe T., Bouhenni R. (2014). Proteomic analysis of keratitis-associated Pseudomonas aeruginosa. Mol. Vis..

[61] Hilliam Y., Kaye S., Winstanley C. (2020). Pseudomonas aeruginosa and microbial keratitis. J. Med. Microbiol..

[62] Sack R., Sathe S., Beaton A.R., McNamara N., Fleiszig S., Ni M. (2009). Protein array characterization of bioactive proteins secreted by immortalized human corneal epithelium in response to pseudomonas constituents. Curr. Eye Res..

[63] Bouhenni R., Dunmire J., Rowe T., Bates J. (2015). Proteomics in the study of bacterial keratitis. Proteomes.

[64] Guo L., Kong Q., Dong Z., Dong W., Fu X., Su L., Tan X. (2017). NLRC3 promotes host resistance against Pseudomonas aeruginosa-induced keratitis by promoting the degradation of IRAK1. Int. J. Mol. Med..

[65] Hazlett L.D., McClellan S., Somayajulu M., Bessert D. (2021). Targeting Inflammation Driven by HMGB1 in Bacterial Keratitis—A Review. Pathogens.

[66] Taube M., Cendra D.M., Elsahn A. (2015). Pattern recognition receptors in microbial keratitis. Eye.

[67] Sun Y., Karmakar M., Roy S., Ramadan R.T., Williams S.R., Howell S., Shive C.L., Han Y., Stopford C.M., Rietsch A. (2010). TLR4 and TLR5 on corneal macrophages regulate Pseudomonas aeruginosa keratitis by signaling through MyD88-dependent and-independent pathways. J. Immunol..

[68] Ross B.X., Gao N., Cui X., Standiford T.J., Xu J., Yu F.X. (2017). IL-24 promotes Pseudomonas aeruginosa keratitis in C57BL/6 mouse corneas. J. Immunol..

[69] Willcox M.D. (2007). Pseudomonas aeruginosa infection and inflammation during contact lens wear: A review. Optom. Vis. Sci..

[70] Sueke H., Kaye S.B., Neal T., Hall A., Tuft S., Parry C.M. (2010). An in vitro investigation of synergy or antagonism between antimicrobial combinations against isolates from bacterial keratitis. Investig. Ophthalmol. Vis. Sci..

[71] Boyd K., Pagan-Duran B., Pink Eye (Conjunctivitis) American Academy of Ophthalmology. EyeSmart^®^ Eye Health. https://www.aao.org/eye-health/diseases/pink-eye-conjunctivitis-list.

[72] Tian R., Zou H., Wang L., Liu L., Song M., Zhang H. (2020). Analysis of differentially expressed genes in bacterial and fungal keratitis. Indian J. Ophthalmol..

[73] Carnt N.A., Cipriani V., Stapleton F.J., Calder V., Willcox M.D. (2019). Association study of single nucleotide polymorphisms in IL-10 and IL-17 genes with the severity of microbial keratitis. Contact Lens Anterior Eye.

[74] Chidambaram J.D., Kannambath S., Srikanthi P., Shah M., Lalitha P., Elakkiya S., Bauer J., Prajna N.V., Holland M.J., Burton M.J. (2017). Persistence of innate immune pathways in late stage human bacterial and fungal keratitis: Results from a comparative transcriptome analysis. Front. Cell. Infect. Microbiol..

[75] Gowda R.N., Redfern R., Frikeche J., Pinglay S., Foster J.W. (2015). Functions of peptidoglycan recognition proteins (Pglyrps) at the ocular surface: Bacterial keratitis in gene-targeted mice deficient in Pglyrp-2,-3 and-4. PLoS ONE.

[76] Kuo M.-T., Chen J.L., Hsu S.L., Chen A., You H.L. (2019). An omics approach to diagnosing or investigating fungal keratitis. Int. J. Mol. Sci..

[77] Ananthi S., Chitra T., Bini R., Prajna N.V., Lalitha P., Dharmalingam K. (2008). Comparative analysis of the tear protein profile in mycotic keratitis patients. Mol. Vis..

[78] Ananthi S., Venkatesh P.N., Lalitha P., Valarnila M., Dharmalingam K. (2013). Pathogen induced changes in the protein profile of human tears from Fusarium keratitis patients. PLoS ONE.

[79] Kandhavelu J., Demonte N.L., Namperumalsamy V.P., Prajna L., Thangavel C., Jayapal J.M., Kuppamuthu D. (2017). Aspergillus flavus induced alterations in tear protein profile reveal pathogen-induced host response to fungal infection. J. Proteom..

[80] Calvillo-Medina R.P., Reyes-Grajeda J.P., Barba-Escoto L., Bautista-Hernandez L.A., Campos-Guillén J., Jones G.H., Bautista-de Lucio V.M. (2019). Proteome analysis of biofilm produced by a Fusarium falciforme keratitis infectious agent. Microb. Pathog..

[81] Maharana P.K., Sharma N., Nagpal R., Jhanji V., Das S., Vajpayee R.B. (2016). Recent advances in diagnosis and management of Mycotic Keratitis. Indian J. Ophthalmol..

[82] Parthiban N., Sampath N.L., Jeya Maheshwari J., Prajna N.V., Lalitha P., Dharmalingam K. (2019). Quantitative profiling of tear proteome reveals down regulation of zinc alpha-2 glycoprotein in Aspergillus flavus keratitis patients. Exp. Eye Res..

[83] Ranjith K., Kalyana C.S., Adicherla H., Sharma S., Shivaji S. (2018). Temporal expression of genes in biofilm-forming ocular Candida albicans isolated from patients with keratitis and orbital cellulitis. Investig. Ophthalmol. Vis. Sci..

[84] Zhang Q., Zhang J., Gong M., Pan R., Liu Y., Tao L., He K. (2020). Transcriptome analysis of the gene expression profiles associated with fungal keratitis in mice based on RNA-Seq. Investig. Ophthalmol. Vis. Sci..

[85] Zhang Y., Liang Q., Liu Y., Pan Z., Baudouin C., Labbé A., Lu Q. (2018). Expression of cytokines in aqueous humor from fungal keratitis patients. BMC Ophthalmol..

[86] Zhao G., Xu Q., Lin J., Chen W., Cui T., Hu L., Jiang N. (2017). The role of Mincle in innate immune to fungal keratitis. J. Infect. Dev. Ctries..

[87] Cui H., Liu Y., Huang Y. (2017). Roles of TRIM32 in corneal epithelial cells after infection with herpes simplex virus. Cell. Physiol. Biochem..

[88] Berard A.R., Coombs K.M., Severini A. (2015). Quantification of the host response proteome after herpes simplex virus type 1 infection. J. Proteome Res..

[89] Wang L., Wang R., Xu C., Zhou H. (2020). Pathogenesis of herpes stromal keratitis: Immune inflammatory response mediated by inflammatory regulators. Front. Immunol..

[90] Maertzdorf J., Osterhaus A.D., Verjans G.M. (2002). IL-17 expression in human herpetic stromal keratitis: Modulatory effects on chemokine production by corneal fibroblasts. J. Immunol..

[91] Nicoll M.P., Proença J.T., Efstathiou S. (2012). The molecular basis of herpes simplex virus latency. FEMS Microbiol. Rev..

[92] Tormanen K., Allen S., Mott K.R., Ghiasi H. (2019). The latency-associated transcript inhibits apoptosis via downregulation of components of the type I interferon pathway during latent herpes simplex virus 1 ocular infection. J. Virol..

[93] Banerjee A., Schambach F., DeJong C.S., Hammond S.M., Reiner S.L. (2010). Micro-RNA-155 inhibits IFN-γ signaling in CD4+ T cells. Eur. J. Immunol..

[94] Huffaker T.B., Hu R., Runtsch M.C., Bake E., Chen X., Zhao J., Round J.L., Baltimore D., O’Connell R.M. (2012). Epistasis between microRNAs 155 and 146a during T cell-mediated antitumor immunity. Cell Rep..

[95] Anand S., Majeti B.K., Acevedo L.M., Murphy E.A., Mukthavaram R., Scheppke L., Huang M., Shields D.J., Lindquist J.N., Lapinski P.E. (2010). MicroRNA-132–mediated loss of p120RasGAP activates the endothelium to facilitate pathological angiogenesis. Nat. Med..

[96] Jaishankar D., Yakoub A.M., Yadavalli T., Agelidis A., Thakkar N., Hadigal S., Ames J., Shukla D. (2018). An off-target effect of BX795 blocks herpes simplex virus type 1 infection of the eye. Sci. Transl. Med..

[97] Chen L., Pan Z.-Q., Zhai C.-B. (2021). Adenovirus-mediated RNA interference against herpes simplex virus infection in vitro. Folia Histochem. Cytobiol..

[98] Yang H., Yang X., Wang Y., Zheng X., Zhang Y., Shao Y. (2020). Comparative analysis of the tear protein profile in herpes simplex virus type 1 epithelial keratitis. BMC Ophthalmol..

[99] Tumpey T.M., Cheng H., Cook D.N., Smithies O., Oakes J.E., Lausch R.N. (1998). Absence of macrophage inflammatory protein-1α prevents the development of blinding herpes stromal keratitis. J. Virol..

[100] Twardy B.S., Channappanavar R., Suvas S. (2011). Substance P in the corneal stroma regulates the severity of herpetic stromal keratitis lesions. Investig. Ophthalmol. Vis. Sci..

[101] Hurt M., Apte S., Leher H., Howard K., Niederkorn J., Alizadeh H. (2001). Exacerbation of Acanthamoeba keratitis in animals treated with anti-macrophage inflammatory protein 2 or antineutrophil antibodies. Infect. Immun..

[102] Dreyfuss J.L., Regatieri C.V., Coelho B., Barbosa J.B., De Freitas D., Nader H.B., Martins J.R. (2015). Altered hyaluronic acid content in tear fluid of patients with adenoviral conjunctivitis. An. Acad. Bras. Ciências.

[103] Wishart D.S., Feunang Y.D., Guo A.C., Lo E.J., Marcu A., Grant J.R., Sajed T., Johnson D., Li C., Sayeeda Z. (2018). DrugBank 5.0: A major update to the DrugBank database for 2018. Nucleic Acids Res..

